# Fiber Optic SPR POCT: A Novel Nucleic Acid Detection Biosensor for Environmental Viruses

**DOI:** 10.34133/research.0296

**Published:** 2024-01-29

**Authors:** Jing Wang, Zhaojun Duan, Dixian Luo

**Affiliations:** ^1^ Department of Laboratory Medicine, Shenzhen Hospital of Integrated Traditional and Western Medicine, Shenzhen, China.; ^2^ National Institute for Viral Disease Control and Prevention, China Center for Disease Control and Prevention, Beijing, China.

## Abstract

In the post-COVID-19 pandemic era, the long-term surveillance of pathogens is still important. The rapid detection of pathogens facilitates the accurate and convenient real-time monitoring of microbial contamination and improves the management of diseases. Here, a novel surface plasmon resonance (SPR)-based point of care testing (POCT) approach of microorganism nucleic acids with the guidance of CRISPR enzyme is described, including the application of optical fiber-based detection of trace SARS-CoV2 virus in sewage water on SPR and validation of the plasmonic biosensor for the detection of single-nucleotide mutations in natural water samples.

Infectious diseases severely threaten both public health and global biosafety. Pathogens are normally transmitted through air, water, and pollutants [1]. The early detection of viral pathogens in clinical samples through highly sensitive bioassay methods can greatly enhance clinical outcomes and mitigate the socioeconomic impact of these diseases. Conventional biochemical and microbiological methodologies have been extensively utilized for the detection of viral pathogens in clinical samples. Unfortunately, they are often time-consuming and cost-ineffective [2]. Furthermore, the choice of virus detection method depends on several factors, including the nature of the virus, the activity and viability time on different surfaces, etc. However, there has been insufficient focus on sensing pathogenic microorganisms in environmental samples, which contain scarce amounts of genetic material [3].

To address these difficulties, a recent study performed by Li et al. [4] in Shenzhen University demonstrates the first application of optic fiber-based detection of trace SARS-CoV-2 virus in sewage water based on SPR. This work introduces a novel SPR-based POCT of microorganism nucleic acids with the guidance of clustered regularly interspaced short palindromic repeats (CRISPR) enzyme (Fig. 1). An ultrafine plasmonic fiber probe, with a diameter of 125 μm, was synthesized for one-time plug-in assembly into the miniaturized SPR apparatus. The specificity of Cas12a ribonucleoprotein (RNP) guarantees the accuracy of the plasmonic biosensor, and the discriminative capability of single-nucleotide polymorphism endows the biosensor with the unique viral genotyping function. During the sample preparation, the viral genomes are automatically enriched and the nucleocapsid (N) gene is amplified before its reaction with CRISPR protein, i.e., Cas12a. Once the N gene is recognized by Cas12a RNP, its *trans*-cleavage activity is activated, facilitating the breakage of single-stranded DNA exposed on the fiber surface and the release of gold nanoparticles (AuNPs) that have been previously immobilized. These result in a reduction in the characteristic SPR wavelength, and the viral genetic information recognized by CRISPR enzyme is converted into SPR signals that are further amplified by the release of AuNPs.

**Fig. 1.**
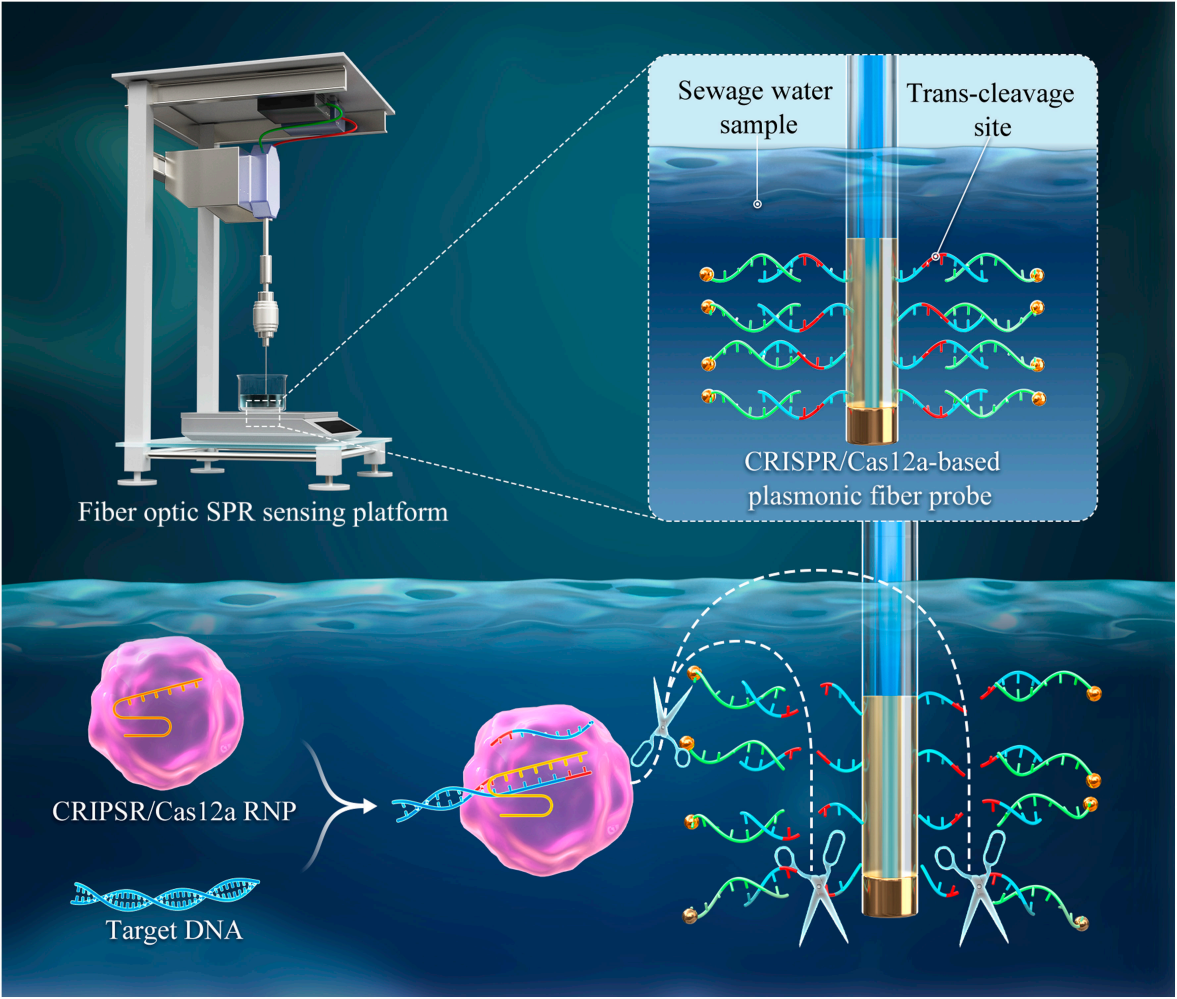
Fiber Optic SPR POCT for nucleic acid detection of environmental viruses.

The single-stranded DNA-modified fiber probe is virus-specific with a limit of detection (LOD) of ~2,300 copies/ml. To test its applicability in environmental samples, Zhang and his co-workers collected sewage water specimens from various sources, including households, natural water bodies, hospitals, airports, etc. These samples have all been examined by fiber SPR, and the data obtained from the biosensor are subsequently validated by quantitative polymerase chain reaction and Cas12a-based colorimetric assays. In particular, the performance of one Cas12a RNP targeting L981F mutation with the plasmonic biosensor has been validated, indicating the fast and successful detection of single-nucleotide mutations in natural water samples.

The end of the COVID-19 pandemic has been declared by the World Health Organization. However, efforts are still required to realize the long-time surveillance of pathogen and prevention of disease spread. Due to the urgent needs for rapid detection of pathogenic viruses during previous pandemics viruses, the CRISPR-Cas9 system has already been employed for fast, precise, and cost-effective nucleic acid detection [5]. Previous research on CRISPR-Cpf1 (Cas12a) has highlighted the potential of the CRISPR-Cas12a system for simultaneous cleavage of both *cis* (target)- and *trans* (non-target)-DNA, utilizing sticky-ends 5 to 7 bp. This system holds promise for the development of versatile electrochemical biosensing platforms (E-CRISPR) that can verify infections caused by single-stranded and double-stranded DNA virus [6]. Specific High-sensitivity Enzymatic Reporter unLOCKing (SHERLOCK) and DNA Endonuclease Targeted CRISPR Trans Reporter (DETECTR) are also diagnostic techniques based on the CRISPR-Cas system and can be used to detect new pathogens even at low concentrations [7]. Several recent reviews discussed SPR-based biosensing for COVID-19 detection [8] and the application of optical fiber SPR sensors in monitoring environmental health with nanocomposite thin films [9]. Fiber-based SPR sensors have also been utilized in the monitoring of therapeutic antibody levels in the bloodstream. This application is particularly valuable due to the substantial variability observed among individuals. By employing fiber SPR sensors, clinical decision-making can be optimized, ultimately leading to improved patient outcomes [10]. Furthermore, a study has presented the development of a cost-effective, optical fiber SPR sensor, enabling the construction of a compact remote sensing system, and extensively characterizing the binding of infliximab to an immobilized anti-infliximab antibody on the surface of the fiber SPR sensor [11]. The portable device is in a simple and transportable device suitable for easy, rapid, and cost-effective point-of-care analysis. In contrast to conventional SPR devices, the portable device boasts compact dimensions, with a maximum width of 10 cm, a maximum length of 35 cm, and a maximum height of 4 cm. Furthermore, there is potential for further reduction in the size of the spectrometer and light source. In the post-COVID-19 pandemic era, the portable biosensors can be widely set up adjacent to drainage outlets in large municipalities for in situ monitoring pathogens. Their ease of assembly and minimized space occupancy reduce the manufacturing cost and labor time consumption. Regarding the frequent outbreak of pathogenic microorganisms, crRNAs can be designed by bioinformatic software and tested in parallel under high-throughput screening. The assembled Cas12a RNPs work collaboratively with the biosensor for the fast and sensitive detection of pathogens. This system has also exhibited superb monkeypox testing ability in a recent study, with an LOD of F8L gene down to the aM concentration level [12]. Besides the sensing of pathogens, the application of the biosensor can be extended to cancer diagnosis or examination of environmental pollution due to the recognition versatility of nucleic acids by CRISPR enzymes. In the future, analytes like cancer microRNAs, cell-free DNAs, and heavy metal ions can all be directly or indirectly recognized by specific Cas12 RNPs, and the signals are then converted and amplified by the biosensor, which may greatly promote cancer diagnosis and environmental protection. In conclusion, this work establishes a potential platform for tomorrow’s accurate and convenient real-time monitoring of microbial contamination in environmental samples.
